# Isolated Penile Fournier's Gangrene: A Rare Case

**DOI:** 10.7759/cureus.7953

**Published:** 2020-05-04

**Authors:** Orkun Batmaz, Murat Ucar, Ahmet E Caylan, İsmail B Gök, Veli Vural

**Affiliations:** 1 Urology, Faculty of Medicine, Akdeniz University, Antalya, TUR; 2 General Surgery, Akdeniz University Hospital, Antalya, TUR

**Keywords:** penis, fornier's gangrene, debridement

## Abstract

Fournier’s gangrene is necrotizing fasciitis involving the penis, scrotum, or perineal region. This condition is associated with a high mortality rate and requires aggressive debridement of necrotic tissues. It is mainly seen in elderly, immune-suppressed, diabetic patients, or patients with long-term urinary catheterization. Isolated penile involvement is very rare due to the rich blood supply of the organ and is thought to be induced by penile trauma with partial involvement of the urethra.^ ^In this study, we present a patient with isolated penile and urethral necrosis rather than scrotum and perineum and emphasize its gastrointestinal source.

## Introduction

Fournier’s gangrene is a multibacterial infection of the genitalia. Surgical debridement is very crucial for treatment in most cases, and delay in management is associated with an increased mortality rate [1]. The affected population typically includes elderly, diabetic, and malnourished males. Owing to the rich blood supply to the penis, isolated penile involvement in Fournier's gangrene is not commonly seen, and almost all cases are associated with penile trauma and prolonged urinary catheterization [2].

## Case presentation

A 70-year-old male patient presented to the emergency department because of poor general and hemodynamic status, pain, and blackish discoloration of the penis. He was diagnosed with anal canal adenocarcinoma and underwent neoadjuvant radiotherapy five years ago. After neoadjuvant therapy, he refused surgery and follow-up visits.

The patient was cachectic and showed signs of poor self-care. He was never checked after pelvic radiotherapy, and as we know, he did not have any other comorbidities except the aforementioned carcinoma. His blood glucose was 106 mg/dL and serum creatinine was 3.89 mg/dL (basal serum creatinine was normal one year ago).

**Figure 1:**
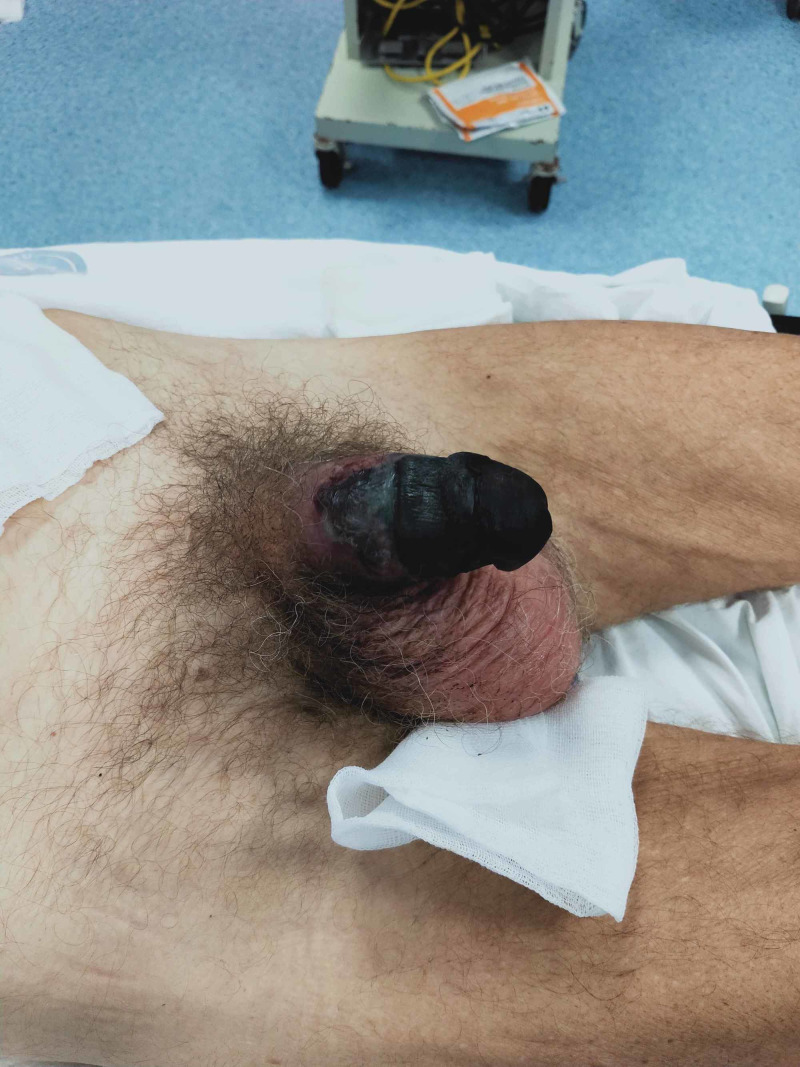
Isolated necrosis of the penis

The urogenital examination revealed the penis was malodorous. The penis was necrotic up to the penoscrotal margin in ventral and midshaft in the dorsal side (Figure 1).

Scrotum and perineum were preserved. The abdominal examination was normal. The anal canal was very hard and tender with palpation, and there was no bloody discharge and abscess formation. 

**Figure 2:**
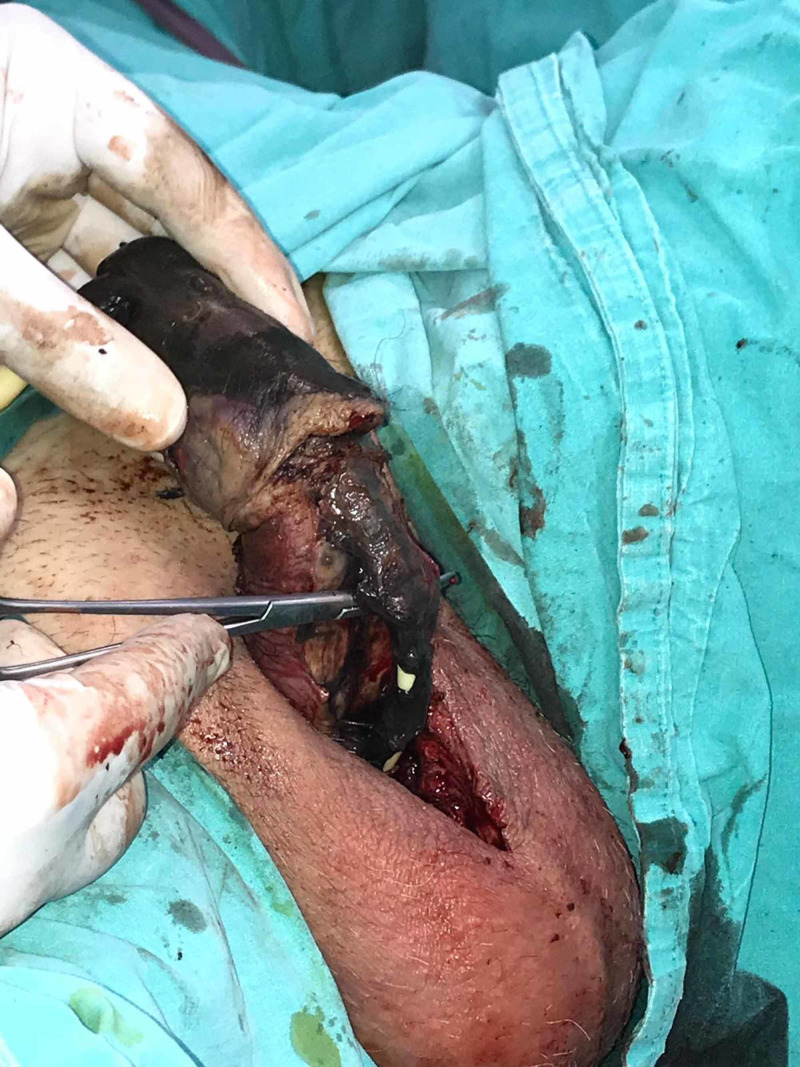
Necrosis extended to urethra

Emergent exploration and debridement were performed. Along the necrosis margin, a circular incision was made and the pus drained and sampled for culture. Corpora cavernosa and urethra were necrotic down to the pubic rami (Figure 2).

Rectoprostatic fascia was mostly necrotic. Rectum mucosa was viable. Scrotum, testicles, and spermatic cords were spared, but the left tunica vaginalis was partially involved. Penectomy, urethrectomy, and debridement of the necrotic tissues were performed. An indwelling cystostomy catheter was placed, and the urine was macroscopically clear without intestinal content, blood, or debris and so we ruled out a fistula. After the surgery, the patient was taken to the intensive care unit, and wide-spectrum antibiotics were started. Penile swab culture resulted in *Klebsiella pneumoniae* spp, and pathological examination revealed chronic granulation tissue with acute onset and severe necrosis.

**Figure 3:**
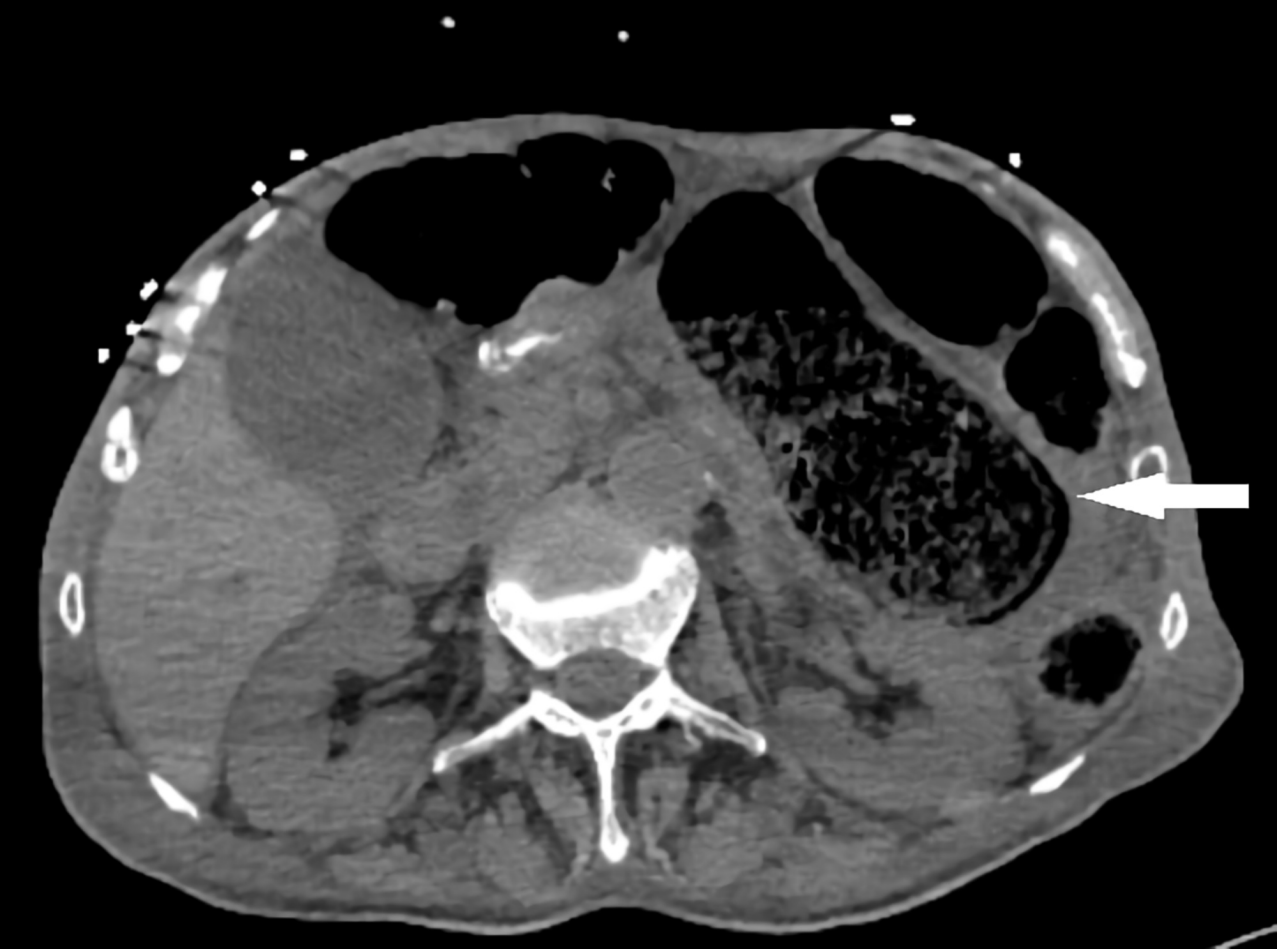
Dilated colon in computed tomography

On the second postoperative day, the patient was still unstable and intestinal content was apparent in the debrided scrotal region. Abdominopelvic CT scan revealed dilatation of descending and sigmoid colon and suspicious fistula tract from the lateral wall of the rectum to the perineum (Figure 3). After surgical consultation, an emergency laparotomy was planned.

**Figure 4:**
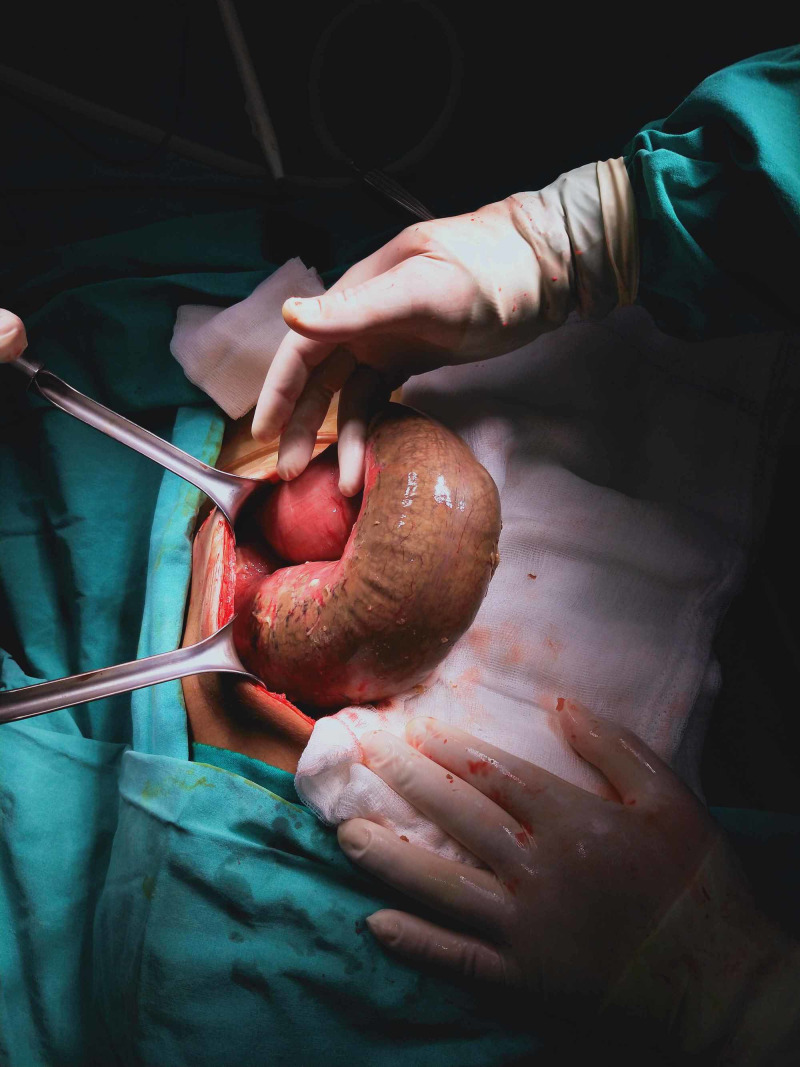
Dilated and necrotic sigmoid colon

After gaining access to the abdomen, purulent fluid was aspirated from the abdomen without any apparent perforation. Multiple liver metastases and peritoneal implants were seen. The sigmoid colon was seen as dilated and necrotic (Figure 4).

However, the mesenteric arterial flow was palpated normal without any signs of vascular occlusion. The necrotic segment was resected, and the distal and proximal segments were constructed as double-barrel colostomy.

On postoperative day four, new gangrenous tissues were seen on testicles, and Doppler ultrasonography failed to show any signs of perfusion. A bilateral orchiectomy was performed.

Although source control was achieved with aggressive debridement, daily careful wound care, and wide-spectrum antibiotherapy, the patient died due to a respiratory infection.

## Discussion

Fournier’s gangrene is a type of necrotizing fasciitis affecting the genitourinary region. The majority of cases are polymicrobial and require emergent debridement and wide spectrum antibiotic treatment. Colorectal sources (30% to 50%), urogenital sources (20% to 40%), cutaneous infections (20%), and local trauma are the main causative agents for Fournier’s gangrene. It is usually seen in immunocompromised like patients with diabetes, end-stage renal disease, and malignancy [1]. Our patient had untreated anal canal adenocarcinoma, and he had undiagnosed multiple metastases, and the mass effect of carcinoma could be the cause of the rectum wall necrosis and following scrotal fistula.

Aggressive debridement of the necrotic tissues until the viable tissues are seen is the standard approach for Fournier’s gangrene [2]. Careful wound care and wide-spectrum antibiotic treatment for both aerobic and anaerobic microorganisms, maintaining normoglycemia, and improving nutritional status are vital, especially in the early period after debridement.

Due to the rich vascular supply from the bulbourethral artery, isolated penile involvement is a very rare form of Fournier’s gangrene. The underlying causes are mostly idiopathic. Penile trauma and urethral stricture were suspected in two cases [3]. Penile injection of cocaine and human bite were reported in another study [4-5].

Panurethral necrosis was rarely reported. Long-term urethral catheterization of an immunocompromised patient was considered a risk factor in the reported cases [1-6].

Usually, corpora cavernosa and tunica albuginea are spared in penile Fournier’s Gangrene, and patients sometimes require partial or total penectomy [6]. In our case, the glans, corpus spongiosum, and corpora cavernosa were necrotic and needed total excision.

Our case is unique as the gangrene originated from the rectoscrotal fistula and isolated penile involvement and necrosis of the whole urethra to the prostatic margin as mentioned above.

A fistula may be secondary to previous radiotherapy or the carcinoma itself. Colonic obstruction may precipitate the passage of the intestinal content to the scrotum.

## Conclusions

Fornier's gangrene can be a life-threatening condition, especially in elderly and immunosuppressed patients. Rapid and aggressive debridement is the main keystone in treatment. Penile and urethral involvement is rare but may require investigation gastrointestinal sources, especially in the absence of trauma. During the presentation of Fournier’s gangrene, immediate abdominal imaging is needed when a gastrointestinal source of infection is suspected. Diagnostic laparotomy may help disease management. Even in delayed presentation, adequate source control is achievable.
